# Supplementary figures and images for: Genome-based development of 15 microsatellite markers in fluorescent multiplexes for parentage testing in captive tigers
Source: PeerJ. 2020 May 6;8:e8939. doi: 10.7717/peerj.8939 (PMC7210807; doi:10.7717/peerj.8939)

A

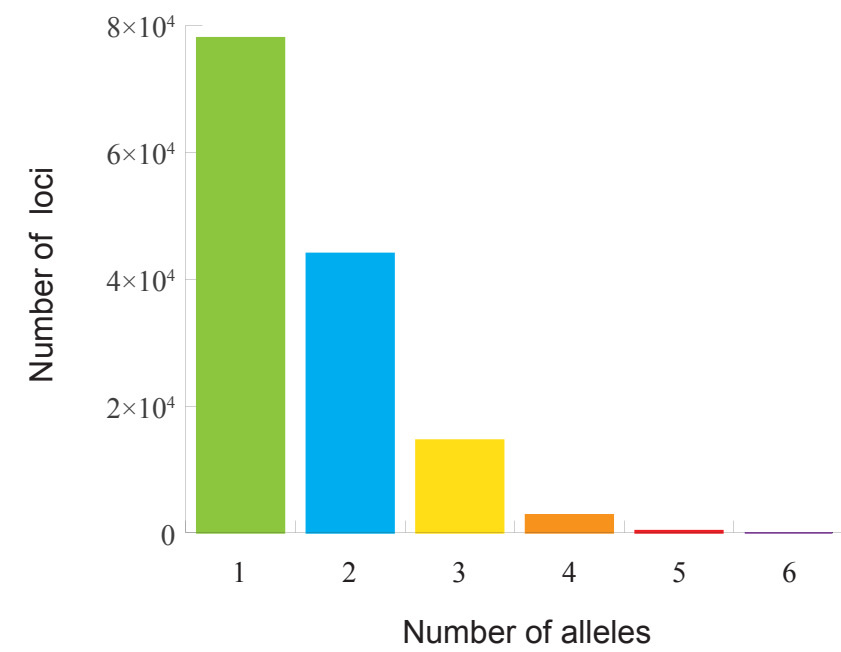

B

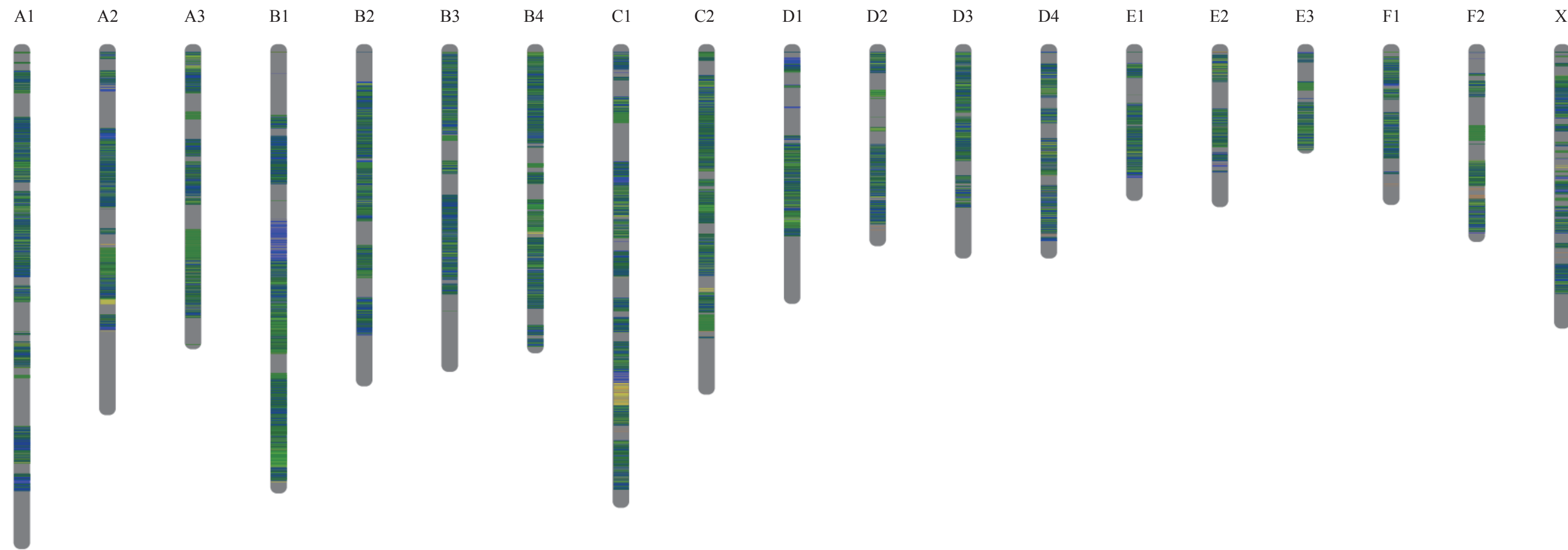

Supplement: Supplemental Information 1 — (A) Distribution of STR loci along different allele number (from 1 to 6). (B) Tiger STR loci and their polymorphisms on chromosomes. Different colors denote STR loci with different allele number according to Fig. 2A. [file peerj-08-8939-s001.pdf]

F-Primer

└─ 5' GAGAGGCAARTAGGAGTGTGC 3'

R-Primer

└─ 5' TTCAAGATGTTTCTCAGTCC 3'

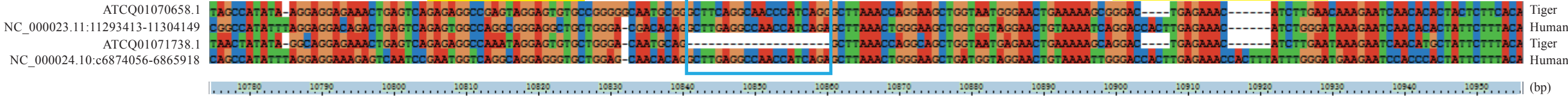

Supplement: Supplemental Information 2 — The 20 bp deletion polymorphism in tiger sequence is boxed in blue. The yellow rectangle marks the sequence region of the designed primers. NC_000023.11:11293413-11304149 and NC_000024.10:c6874056-6865918, human chromosome; ATCQ01070658.1 and ATCQ01071738.1, tiger scaffolds. [file peerj-08-8939-s002.pdf]
