# Supplementary material for: Genome-based development of 15 microsatellite markers in fluorescent multiplexes for parentage testing in captive tigers
Source: PeerJ. 2020 May 6;8:e8939. doi: 10.7717/peerj.8939 (PMC7210807; doi:10.7717/peerj.8939)
Supplement: Supplemental Information 3 — SIP stands for the sex information provided by the zoo staff. SII stands for the sex information identified by the TAMEL locus. The numbers above the peaks are length scales based on the internal size standard (bp). The numbers below the peaks are the fragment lengths of PCR amplifications (bp). [file peerj-08-8939-s003.pdf]

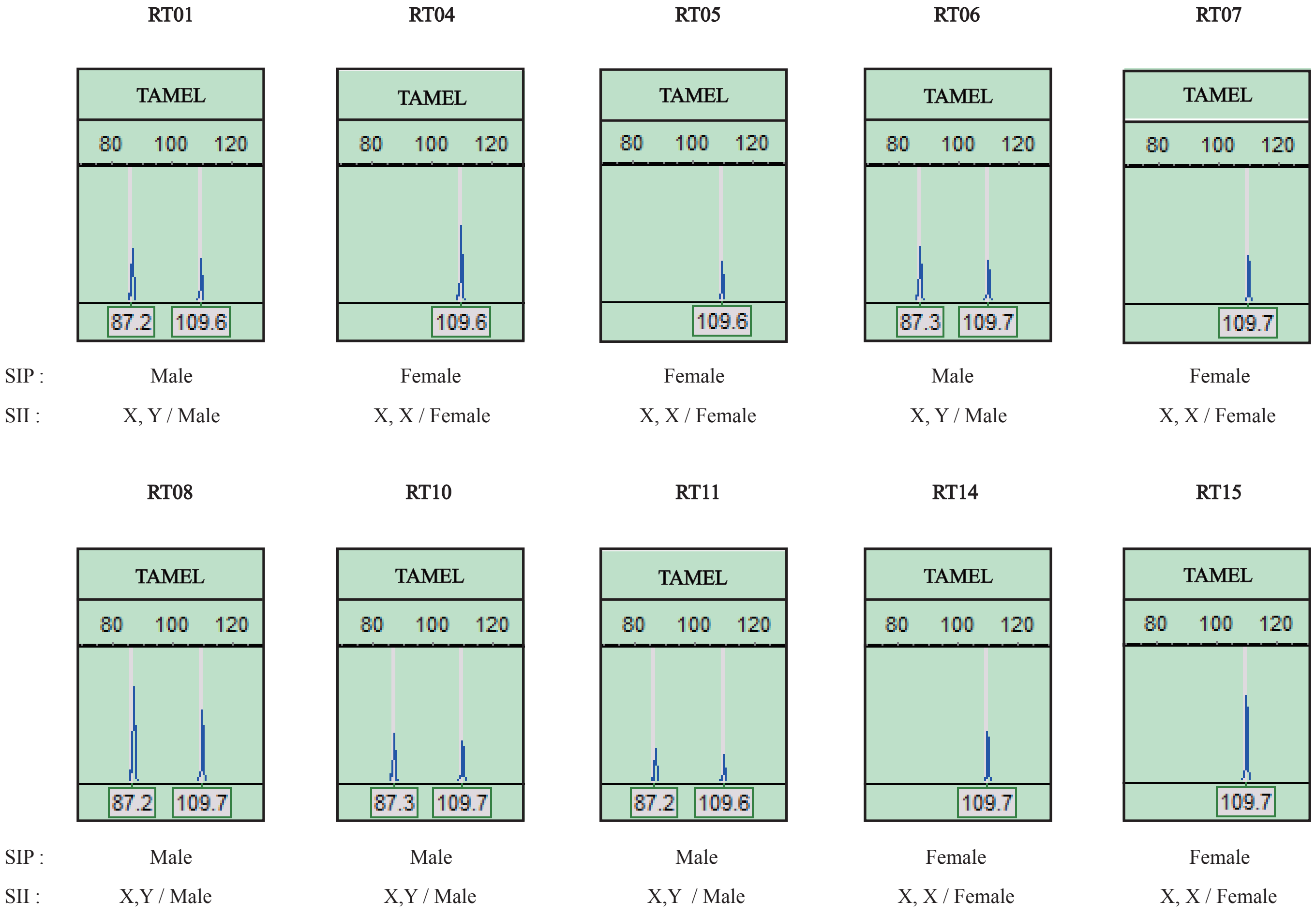
RT01
TAMEL
80 100 120
87.2 109.6
RT04
TAMEL
80 100 120
109.6
RT05
TAMEL
80 100 120
109.6
RT06
TAMEL
80 100 120
87.3 109.7
RT07
TAMEL
80 100 120
109.7
SIP : Male Female Female Male Female
SII : X, Y / Male X, X / Female X, X / Female X, Y / Male X, X / Female
RT08
TAMEL
80 100 120
87.2 109.7
RT10
TAMEL
80 100 120
87.3 109.7
RT11
TAMEL
80 100 120
87.2 109.6
RT14
TAMEL
80 100 120
109.7
RT15
TAMEL
80 100 120
109.7
SIP : Male Male Male Female Female
SII : X,Y / Male X,Y / Male X,Y / Male X, X / Female X, X / Female
